# Role of district health management teams in child health strategies

**DOI:** 10.1136/bmj.k2823

**Published:** 2018-07-30

**Authors:** Tanya Doherty, Nhan Tran, David Sanders, Sarah L Dalglish, David Hipgrave, Kumanan Rasanathan, Thiagarajan Sundararaman, Rajani Ved, Elizabeth Mason

**Affiliations:** 1 Health Systems Research Unit, South African Medical Research Council, Cape Town, South Africa; 2 School of Public Health, University of the Western Cape, Cape Town, South Africa; 3 School of Public Health, University of the Witwatersrand, Johannesburg, South Africa; 4 Alliance for Health Policy and Systems Research, World Health Organization, Geneva, Switzerland; 5 Department of Paediatrics and Child Health, University of Cape Town; 6 Department of Maternal, Newborn, Child and Adolescent Health, World Health Organization, Geneva, Switzerland; 7 Health Section, Unicef, New York, USA; 8 School of Health Systems Studies, Tata Institute of Social Studies, Mumbai, India; 9 National Health Systems Resource Center, New Delhi, India; 10 Institute for Global Health, University College London, London, UK; Correspondence to: T Doherty tanya.doherty@mrc.ac.za

## Abstract

Well functioning district health systems are essential for planning and implementation of health services, and their efforts are key to improving quality of care and achieving health goals, say **Tanya Doherty and colleagues**

The district health system usually includes a network of hospitals and primary healthcare facilities catering to a specified population, under the guidance of district health management teams. This structure was developed to facilitate the management and implementation of primary healthcare.^1^ ^2^ The management teams’ responsibilities include planning and budgeting, human resource management, monitoring service quality, and ideally allocating resources to most effectively meet local population needs.

Recent evidence suggests that poor performance of district management teams may hinder scaling up of proved health interventions in low and middle income countries.^3^ Weak performance can result in poor coordination of health service delivery, insufficient funding for affordable and effective health programmes, and inadequate resources for health workers to provide high quality healthcare. Resource related consequences of weak management include running out of medicines, delayed repair of broken equipment, and health worker absenteeism. Poor management can adversely affect health workers’ motivation and may contribute to people moving from the public to the private sector.^4^ This may reduce the quality of care at health facilities or reduce demand for health services as users of public facilities come to know that certain facilities provide suboptimal services and choose to seek alternative care from traditional medicine providers or refrain from seeking healthcare other than for emergencies.^5^ ^6^

Health sector reforms in many low and middle income countries have promoted a shift towards greater decentralisation of responsibilities to districts.^7^ However, this shift has often not been accompanied by commensurate attention to management capacity, allocation of resources, or autonomy to enable effective priority setting, planning, and implementation.^8^ As a result many district management teams cannot function as intended, and the perceived benefits of decentralisation—namely, accountability and responsiveness to communities and their specific context and improved access to quality services and health outcomes—have not been realised.^9^ For example, district managers who identify a need for supervision of trained child health workers at facilities may have insufficient access to funds to provide such supervision.

We report the findings of the 2016 strategic review of Integrated Management of Childhood Illness (IMCI), focusing on district level management of child health services.^10^ ^11^ Using country examples, we present lessons learnt and identify options for improving the effectiveness of district health management teams, with respect to implementation of child health services, particularly IMCI. We derived data from four main sources: the IMCI implementation survey of 95 five countries,^10^ 20 interviews with global informants, nine in-depth country assessments, and eight short vignettes of child health strategies being effectively implemented. Although the review’s focus was IMCI, the findings presented are broadly relevant to district implementation of primary healthcare.

## Do district teams have the skills, resources, and authority to manage?

Interviews with key informants and country case studies reaffirmed the critical importance of strong management teams for the functioning of district health systems; yet a commonly reported challenge was an insufficient focus on district management capacity. The district management teams are responsible for overseeing all health services provided in their catchment, including child health services (such as immunisation, growth monitoring, and assessment and treatment of sick children), yet lacked the skills and authority to effectively prioritise, plan, implement, and monitor services. This could result in problems such as shortages of antimalarials and other essential lifesaving drugs.

Strengthening district management capacity was arguably a neglected element of the introduction of IMCI. District managers received the same IMCI training as frontline health workers, despite their primary function being management not clinical care. In 2009 WHO launched a training course aimed at managers of child health focused programmes,^12^ designed to improve knowledge and skills in developing the implementation plans using local data and to implement activities and monitor progress. The IMCI implementation survey found that this training had been introduced in less than half (47%) of the countries because financial resources were limited and clinical training was prioritised over management training.

As well as management skills, district management teams need adequate resources to run the strategy effectively. Financing of IMCI at district level was found to be a major challenge, with insufficient budget for clinical training being the second largest obstacle (after staff turnover) to implementation (reported by 82% of countries in the IMCI implementation survey). With regard to funding for IMCI introduction at primary care facilities, only 32% of countries reported government to be the primary funder of training, with most of the cost being provided by bilateral and multilateral donors. 

Reliance on implementation partners and donors for funding of training means that it is linked to grant periods and risks activities stalling between grants or partners moving out of districts without government funding to take over expenses. It may also discourage the allocation of local resources to fund such training. For a child health strategy such as IMCI, which requires refresher or ongoing training to maintain quality or account for staff turnover, in conjunction with regular on-site supervision, gaps in training and supervision can affect health outcomes.

The absence of authority to allocate very limited resources has led to heavy dependence on partners for technical and financial support (box 1). A common theme to emerge from those interviewed was lack of ownership of IMCI at district level. In contrast, ownership of the Expanded Programme on Immunisation (EPI), implemented as a vertical intervention with dedicated funding and lines of responsibility and reporting at district level, was described to be greater.

Box 1 **Country experiences: district ownership of and resources to manage IMCI**

“It should be a programme of district managers. They do feel ownership of EPI and nutrition programmes, but not for IMCI (technical officer, Saving Newborn Lives, Bangladesh)”.

“At state level, most of these interventions are partner driven.” (technical officer at international agency, Nigeria)

“At the ground level, regular recording was hampered by availability of forms and registers, and by absence of supervision. At the district and state level, since the indicators to monitor the programme performance are not included in the HMIS [health management information system], the information flow was not regular and it is very easy to miss out something that is not regularly measured and monitored” (civil society representative, India)

“A costed child health plan exists, but government did not allocate money for capacity building so relies on partners. States and districts are encouraged to use their own funds—ie, World Bank money” (senior ministry of health official, Myanmar)

“Insufficient support from the partner(s), out-of-stock medicines and other inputs, and a mobile workforce are all challenges the [district] management teams must constantly face. Solutions often depend on the partner.” (DRC country assessment)

“The focus must now turn to leadership and capacity at the woreda [district] level, particularly in terms of enhancing planning and management skills and building toward ownership of IMCI and other child survival programming.” (Ethiopia country assessment)

## Managing delivery in context of competing priorities

Management teams have to balance many competing priorities and programme areas, some of which are strongly driven by higher levels of the health system, are better resourced, and have more stringent reporting than IMCI. Several of these newer programmes are meant to be complementary to IMCI, such as the Global Action Plan for Pneumonia and Diarrhoea (GAPPD) or integrated community case management (iCCM), but often receive greater priority because of vertical funding and donor support (box 2).

Box 2 **Countries’ experiences: competing priorities at district level**

“The Global Action Plan for Pneumonia and Diarrhoea can complement and strengthen IMCI … it should not be implemented as a standalone intervention. It seems to be competing rather than complementing IMCI” (academic researcher, Bangladesh)

“Stakeholders including Gavi and the Global Fund had sought to improve procurement and supply chain issues for individual medicines or classes of drugs, whereas there are hundreds of drugs I want to get right” (government policymaker, Ethiopia)

“Since the launch of IMCI, the country’s child health strategy has come a long way, and along the journey various competing programmes have stepped in. In some ways, it has led to confusion at the grass-root level. For instance, the antibiotic policy for home-based newborn care and IMCI is at variance” (former government official, India)

“There is considerable enthusiasm for implementing the community component. There are directives, rules, and tools for implementation and monitoring the progress of local volunteers. Sick newborns are routinely referred once warning signs have been detected. Unfortunately, registered nurses working in health centres are not trained to manage these cases as initially planned to ensure continuity of care.” (DRC country assessment)

Simultaneous delivery of different strategies and programmes can create confusion among frontline health workers—for example, when clinical recommendations on antibiotic use are inconsistent, or when there is poor coordination between frontline and referral care. This can reduce the quality and continuity of care for sick children.

A further challenge, however, is the lack of sustained commitment to initiatives which receive much support at the planning and launch stage but inadequate funding for ongoing implementation. This has led to efforts by the multilateral funders (Gavi, the Global Fund, and the Global Financing Facility) to better engage country financial authorities in forward planning and mobilisation of domestic resources for public health services. This remains a work in progress.

## Country solutions

Box 3 gives examples of activities to strengthen district management identified from the country assessments in Nepal and the Democratic Republic of the Congo (DRC). In Nepal the focus was on capacity building specifically for IMCI, and in the DRC a more general approach to health management coaching was applied.

Box 3 **Examples of strategies to strengthen management capacity**

**Nepal**

The government of Nepal substituted two days of IMCI training with two days of IMCI management training for programme managers focusing on planning, supervision, and monitoring of IMCI. They targeted staff involved in the management of district supply chain and health post management and covered logistics, situation analysis, and managerial skills. The training was also given to supervisors of community health workers and community health volunteers implementing community IMCI to ensure consistency in management and supervision of facility and community based IMCI.^13^

**Democratic Republic of Congo**

The Ministry of Health decentralised healthcare provision to provinces in 2006. New positions called provincial technical advisers were created. The advisers facilitate improvement of service delivery within two or three health districts by providing technical guidance and coaching to district management teams.^14^ This approach forms part of the performance based financing system and includes corrective actions when performance indicators are poor. For example, coaching would be provided to reduce user fees if performance indicators showed that patients’ access to health facilities was compromised because of financial barriers.^15^

In addition to strengthening management capacity, ownership of IMCI was improved by appointing people within the management team to be district IMCI coordinators, charged with overseeing the strategy. The appointment of national, regional, and district IMCI coordinators is a core part of the IMCI strategy, and the IMCI implementation survey found that 68% of countries had made such appointments at regional or district level. Country assessments reaffirmed the importance of these positions and identified important functions. Key informants in Nepal suggested that the district IMCI coordinators should maintain records of IMCI trained staff, so that on-site training of any new staff could be arranged. The coordinator would also be responsible for supervising health workers at facility level. During a group interview with non-governmental programme managers and academics in Bangladesh, one person said: “Transfer of government employees is a normal phenomenon. Therefore we should make efforts to incorporate these things in the system so that the mid-level managers in the districts are trained and they are asked to supervise, and this is done on a regular basis.”

Kazakhstan has IMCI coordinators at district level, and monitoring of IMCI implementation is the responsibility of the chief district paediatrician, a senior manager accountable for child health services. Furthermore, IMCI training is a criterion for facility accreditation, which ensures high coverage of IMCI and maintenance of quality child healthcare. Kazakhstan is one of the few countries that has successfully scaled up coverage of IMCI nationally. It has created 16 regional training centres for health workers, with training fully funded by the public health budget.

Tanzania and Peru are two countries that have devolved budgeting and spending for certain health services to subnational levels. Tanzania introduced a mandatory separate budget line for district level IMCI.^16^ This decentralisation of budget allocation, together with the presence of stable district management teams was found to have an important positive influence on IMCI implementation.^17^ Peru has devolved autonomy over budgeting for all maternal, newborn, and child health services to regional level, based on nationally agreed coverage and impact indicators (box 4).

Box 4 **Peru’s decentralised budgeting and health spending**

- The Ministry of Economy and Finance introduced results based budgeting, in coordination with other sectors, for the strategic maternal-neonatal and nutrition programmes in 2007
- Results are measured through a monitoring and evaluation system that collects data quarterly and through information from the most recent demographic and health survey
- Each programme has a defined budget that is divided up by region
- It includes per capita costing for each service provided at health facilities, taking into account specific needs in terms of health workforce, equipment, supplies, training, and supervision
- The approach gives greater autonomy to regional managers, who take ownership for increasing efficiency and equity in the coverage of these interventions

Comprehensive, multisectoral district health plans should form the basis for implementation of integrated services at facility and community levels. Donors, implementing agencies, and community stakeholders should be involved in developing plans to ensure they meet everyone needs and mitigate competing priorities. Strengthening integrated planning is particularly important given that most training costs for IMCI are met by donor agencies, which have their own reporting requirements and timeframes. We identified several examples of countries that have tried to strengthen district planning processes, including Egypt, which introduced district planning specifically for IMCI, and Nepal, which adopted a broader focus.

**Fig 1:**
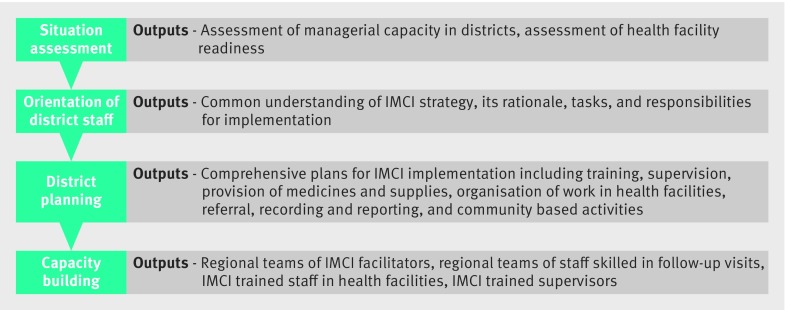
Role of district management teams in systematic scaling up of IMCI in Egypt (adapted from Rahka et al^18^)

Egypt established district IMCI coordinators to lead implementation of the strategy and allocated a government budget. An initial situational assessment of district managerial capacity, health facility readiness, and community resources informed district planning and capacity building.^18^ Well defined IMCI indicators were developed and monitored. A regional systematic approach for scale-up, with sequential steps and quality criteria for each step, was adopted (fig 1). The approach enabled Egypt to expand IMCI to reach 84% of public health facilities over eight years, and a retrospective analysis of trends in under 5 mortality found that the rate of reduction doubled after IMCI was implemented.^18^

Strengthening of district health systems in Nepal is a broader process, with the management of peripheral government health facilities being decentralised to local health facility operation and management committees. The process began in 2002. The committees comprise community representatives, including members elected by the village development committee, school teachers, community health volunteers, and local women, thereby acting as an important bridge between the community and health facility.

Nepal refined and consolidated this approach through its health facility management strengthening programme, developed in 2009. It focuses on intensive capacity building and aims to strengthen the management efficiency and governance of health facilities. The approach involves two years of technical support to health facilities from field officers (one year intensive implementation and one year limited technical support).

A 2012 evaluation of the programme reported several benefits, including increases in community participation, prioritisation of health issues, development of action plans based on local health needs, sharing of decisions with the community, and increased mobilisation of local resources for health.^19^ Despite these advances, decentralisation in Nepal has only been partially successful. The programme continues to receive high level investment from implementing partners with limited devolution of autonomy in budgeting, and overall Nepal’s health system remains highly centralised, exacerbated by ongoing political instability. The impact of recent further experiments with financial decentralisation in some districts remains to be seen.^20^ ^21^

In addition to these country examples, global work is ongoing to improve learning processes within districts and provinces during implementation. These initiatives, led by the Alliance for Health Policy and Systems Research at WHO, are aimed at embedding implementation research as part of routine implementation and engaging programme managers and other implementers as key drivers of the research. Early outcomes indicate improvements in the capacity of the programmes to tackle common implementation barriers and bottlenecks as well as greater use of routine data and other evidence in decision making.^21^ ^22^ These approaches have potential value in enhancing IMCI.

## Other approaches

Our review highlighted the need to improve the capacity of district management teams to manage district health services. An alternative solution might be to reconsider the role of the teams in strengthening the delivery of healthcare and whether other structures might be more effective. The main alternative would be to contract district management positions to private and non-government organisations. Although this model is consistent with a growing practice by donors to support the hiring and retention of qualified managers on behalf of governments in low and middle income countries, it does not enable sustainable strengthening of management capacity within health systems as these positions may cease or change when donor commitments end.

Another approach to strengthening district management that is being tested in several countries with support from the World Bank is performance based financing. This approach aims to improve health system performance by giving health workers or health facilities incentives to reach predetermined targets and outcomes. Many initiatives focus on maternal and child health targets, although these are often not management indicators (eg, completeness of health data reporting or drug stock management), even though reaching clinical targets necessitates improvements to planning and health system management. Evidence of the effectiveness of performance based financing is mixed,^23^ and it has recently been challenged as a “donor fad” suffering from incomplete implementation and perverse effects.^24^ Further research is required to determine whether this approach could be maintained through domestic funding and whether it leads to system-wide, long term improvements in health system performance.

## Conclusions

Pressure to achieve global goals has resulted in many vertical strategies and initiatives being thrust onto districts without the required authority or capacity for prioritising and planning services, allocating resources, or even sufficient resources on their own.^25^ The pressure to implement and scale up programmes without sufficient attention to district health system capacity and resources has contributed to poor ownership of programmes by district management teams and donor driven planning and prioritisation.^26^ The repeated intrusion of new vertical programmes and initiatives, some of which overlap with IMCI, can be disruptive and compete for the time and attention of district teams rather than building on an integrated service delivery platform, such as IMCI. 

There have been few sustained attempts at strengthening district management teams across programme areas, even though this is recognised to be critical to success. Furthermore, strengthening is not a one-off process but requires long term commitment through continued mentorship and support from dedicated district supervisors or peer supervisors at facility level.^27^ ^28^

IMCI could be used as an entry point for building the capacity of district management teams, including to manage programmes that are horizontally integrated.^29^ Strengthening the skills of teams in evidence informed planning and monitoring is essential to achieve global health goals such as universal health coverage, since primary healthcare is mainly implemented and managed at district level.

**Key messages**

- Districts, led by district health management teams, are responsible for implementation and management of primary healthcare
- Health reforms have led to decentralisation of responsibility for service delivery to districts, often without commensurate authority and resources
- District teams often have insufficient management skills or resources to effectively implement the wide scope of health programmes they oversee
- Specific management training and coaching, integrated district planning processes, and decentralised allocation of resources are essential for effective management
- Embedded implementation research and district-to-district learning will facilitate testing of strategies and enable local solutions to emerge

## Notes

See www.bmj.com/child-health for other articles in the series.

Provenance and peer review: Commissioned; externally peer reviewed.

This article is part of a series based on findings from a global review of Integrated Management of Childhood Illness (IMCI) funded by the Bill and Melinda Gates Foundation. Open access fees were funded by the Health Systems Research Unit, South African Medical Research Council. *The BMJ* peer reviewed, edited, and made the decision to publish the article with no involvement from the Medical Research Council.
